# Protocol of the PANCALYZE trial: a multicenter, prospective study investigating the tumor biomarkers CXCR4, SMAD4, SOX9 and IFIT3 in patients with resected pancreatic adenocarcinoma to predict the pattern of recurrence of the disease

**DOI:** 10.1186/s12885-017-3186-8

**Published:** 2017-03-29

**Authors:** Felix C. Popp, Marie Christine Popp, Yue Zhao, Christopher Betzler, Siegfried Kropf, Benjamin Garlipp, Christoph Benckert, Thomas Kalinski, Hans Lippert, Christiane J. Bruns

**Affiliations:** 1 Department of General, Visceral and Cancer Surgery, University of Cologne, Cologne, Germany; 2 Institute for Biometrics and Medical Informatics, Otto-von-Guericke University, Magdeburg, Germany; 3 Department of Pathology, Otto von Guericke University, Magdeburg, Germany; 4 Institute for Quality Control in Operative Medicine, Otto von Guericke University, Magdeburg, Germany; 5 Clinic for General, Visceral and Vascular Surgery, University Clinic Magdeburg, Magdeburg, Germany; 6 Department of General and Visceral Surgery, Vivantes Klinikum im Friedrichshain, Berlin, Germany

**Keywords:** Biomarker analysis, Pancreatic ductal adenocarcinoma, Clinical test development, Prediction of outcome, Personalized therapy, Surgery, Pattern of recurrence, Prognosis of pancreatic cancer, outcome, Immunohistochemistry, Rt.-PCR

## Abstract

**Background:**

Pancreatic ductal adenocarcinoma (PDAC) is one of the most lethal malignancies today with an urgent need for novel therapeutic strategies. Biomarker analysis helps to better understand tumor biology and might emerge as a tool to develop personalized therapies. The aim of the study is to investigate four promising biomarkers to predict the clinical course and particularly the pattern of tumor recurrence after surgical resection.

**Design:**

Patients undergoing surgery for PDAC can be enrolled into the PANCALYZE trial. Biomarker expression of CXCR4, SMAD4, SOX9 and IFIT3 will be prospectively assessed by immunohistochemistry and verified by rt.-PCR from tumor and adjacent healthy pancreatic tissue of surgical specimen. Immunohistochemistry expression pattern of all four biomarkers will be combined into a single score. Beginning with the hospital stay clinical data from enrolled patients will be collected and followed. Different adjuvant chemotherapy protocols will be used to create subgroups. The combined biomarker expression score will be correlated with the further clinical course of the patients to test the hypothesis if CXCR4 positive, SMAD4 negative, SOX9 positive, IFIT3 positive tumors will predominantly develop metastatic spread.

**Discussion:**

Pancreatic cancer is associated with different patterns of progression requiring personalized therapeutic strategies. Biomarker expression analysis might be a tool to predict the pattern of tumor recurrence and discriminate patients that develop systemic metastatic disease from those with tumors that rather develop local recurrence over time. This data might lead to personalized adjuvant treatment decisions as patients with tumors that stay localized might benefit from adjuvant local therapies like radiochemotherapy as compared to those with systemic recurrence who would benefit exclusively from chemotherapy.

Moreover, the pattern of propagation might be a predefined characteristic of pancreatic cancer determined by the genetic signature of the tumor. In the future, biomarker expression analysis could be performed on tumor biopsies to develop personalized therapeutic pathways right after diagnosis of cancer.

**Trial registration:**

German Clinical Trials Register, DRKS00006179.

## Background

Pancreatic ductal adenocarcinoma (PDAC) is the fourth leading cause of cancer related death in Germany and the incidence of PDAC increases constantly in most of the Western countries. Predictions assume that PDAC will become the second leading cause of cancer-related death by 2030 uncovering PDAC to be a serious threat to public health [1]. The 5-year overall survival rate is around 6% making PDAC to one of the most lethal cancers of all known malignancies. Surgical resection remains the only potential curative treatment for PDAC raising the 5-year survival rate up to 14%. However, at the time of diagnosis less than 20% of the tumors are resectable while most are locally advanced or metastasized and thus inoperable.

The reasons for the late time of diagnosis are lack of specific symptoms in the early stage, nonspecific and varying symptoms in the advanced stage as well as rapidly aggressive tumor growth with a high tendency for metastatic spread. Today, postoperative adjuvant chemotherapy (CTx) based on gemcitabine or 5-fluorouracile is standard care of treatment for resected patients. However, despite adjuvant therapy almost 80% of all patients suffer from tumor recurrence within 2 years after resection. With a probability of 20% to 30% tumor recurrence is locally restricted whereas in the majority of patients the tumor systemically spreads to distant sites like liver and peritoneum [2, 3].

According to current clinical guidelines, patients with locally advanced, unresectable non-metastatic pancreatic cancer (LAPC) can receive intensified induction chemotherapy/radiochemotherapy to eventually achieve resectability. This induction therapy selects patients into two groups over time: some tumors stay localized and surgery may be attempted. All other tumors develop chemoresistance and subsequently metastatic disease. Induction therapy might influence tumor biology as tumors can acquire additional pathogenic mutations and epigenetic changes under chemotherapy/radiochemotherapy. However, Krishnan et al. found 50% of 247 patients with LAPC developing metastatic disease under induction radiochemotherapy and 45% of 76 patients under sequential chemotherapy followed by chemoradiotherapy [4]. This observation suggests that the type of induction therapy might not fundamentally influence the type of tumor dissemination and that the type of tumor dissemination (metastatic disease versus localized disease) might be a principal characteristic of pancreatic cancer determined by the initial genetic signature of the tumor. If this basic characteristic could be assessed with biomarker expression analysis at the time of diagnosis tumor treatment could be optimized individually in the future.

After resection, patients with tumors that stay localized over time might benefit from additional local therapies, like adjuvant radiochemotherapy. Patients at risk for developing early metastatic disease might not benefit from surgery at all. To develop individualized therapies for PDAC patients it is necessary to anticipate the clinical course of the disease. With specific biomarkers or a panel of biomarkers it might be possible to predict the pattern of recurrence and distinguish patients who will develop locally restricted disease from those who will suffer from distant metastatic spread. Studies already have been carried out to identify single biomarkers predicting the clinical course of PDAC but none were sufficient to infer treatment schemes for individual patients (for a systematic review, see [5]). Bachet et al. found high CXCR4 expression to be significantly associated with distant recurrence but the sensitivity and specificity of high CXCR4 expression to predict metastatic disease was 55% and 63%, respectively [6]. In this study we will prospectively analyze a panel of 4 biomarkers to predict the clinical course of PDAC after surgical resection. We hypothesize that a combination of biomarkers is superior to a single biomarker in anticipating the individual clinical course. We aim to identify patients who develop metastatic disease with a score derived from biomarker expression analysis. To do so we will analyze tumor samples from pancreatic resections and correlate the combined immunohistochemistry score with the clinical course to test our hypothesis. The 4 biomarkers might help to personalize treatment regimens in the future, thereby extending survival and reducing side effects of pancreatic cancer treatment.

## Design

In this study (see Table 1) we will analyze 4 biomarkers, two established proteins -CXCR4 and SMAD4- that are believed to be related to PDAC outcome and two new biomarkers derived from our own basic research -SOX9 and IFIT3- that were already studied in a small group of patients. We will test the hypothesis that patients with CXCR4^+^SMAD4^−^SOX9^+^IFIT3^+^ primary PDAC will presumably develop metastatic disease. To that end patients with primary PDAC undergoing surgical resection will be included. Likewise patients with locally advanced tumors after receiving neoadjuvant treatment regimens to downsize the tumor can enter this study when tumor resection is performed. Participation in other studies is not an exclusion criterion and patients may enter additional chemotherapy studies that do not interfere with our biomarker analysis.

**Table 1:** World Health Organization Trial Registration Data Set describing the PANCALYZE study

Data category	Information
Primary registry and trial identifying number	German Clinical Trials Register (DRKS) http://drks-neu.uniklinik-freiburg.de/drks_web/ DRKS00006179
Date of registration in primary registry	2014/05/20
Secondary identifying numbers	-
Source(s) of monetary or material support	Department of General, Visceral and Cancer Surgery, University of Cologne, Germany Clinic for General, Visceral and Vascular Surgery, University Clinic Magdeburg, Germany Celgene GmbH, Joseph-Wild-Straße 20, 81,829 München
Primary sponsor	Department of General, Visceral and Cancer Surgery, University of Cologne, Germany Kerpener Str. 62, 50,937 Köln
Secondary sponsor(s)	-
Contact for public queries	Dr. Felix Popp Department of General, Visceral and Cancer Surgery, University of Cologne, Germany Kerpener Str. 62, 50,937 Köln pancalyze@uk-koeln.de
Contact for scientific queries	Dr. Felix Popp Department of General, Visceral and Cancer Surgery, University of Cologne, Germany Kerpener Str. 62, 50,937 Köln pancalyze@uk-koeln.de
Public title	Evaluation of molecular markers to predict the pattern of tumor recurrence and prognosis in pancreatric ductal adenocarcinoma
Scientific title	A multicenter, prospective study investigating the tumor biomarkers CXCR4, SMAD4, SOX9 and IFIT3 in patients with resected pancreatic adenocarcinoma to predict the pattern of recurrence of the disease
Countries of recruitment	Germany
Health condition(s) or problem(s) studied	C25 - Malignant neoplasm of pancreas
Intervention(s)	none
Key inclusion and exclusion criteria	inclusion criteria: patients with primary PDAC undergoing surgical resection exclusion criteria: Patient is inoperable, No tumor samples can be taken, no ductal adenocarcinoma or intraductal papillary mucinous neoplasm (IPMN) in the final histology
Study type	Non-interventional observation
Date of first enrolment	May 2014
Target sample size	450
Recruitment status	Recruiting
Primary outcome(s)	Correlation of biomarker expression and clinical course
Key secondary outcomes	none
Version	2014/05/20 Original Version 1.0

To receive a homogenous patient population we will conduct a multicenter study with a planned short recruitment period. This approach ensures that the patients receive similar postoperative adjuvant treatment protocols and allows a longer follow-up. It is planned to recruit around 410 patients in 14 study centers located in 6 different German states within 3 years.

Patients will be admitted to one of the participating centers and evaluated for study inclusion. If PDAC is suspected and curative surgery is intended the patient can enroll into this study after giving informed consent to the surgeon. Surgery will be performed with no specific requirements from the study protocol according to the German S3-guidelines for PDAC [7]. During surgery peripheral blood will be taken and stored in the biobank to answer possible future scientific questions. After successful resection the pathologist will prepare paraffin embedded tumor samples and samples of adjacent pancreatic tissue from the surgical specimen and send them to the University Hospital Cologne establishing a central biobank. If possible, additional tumor samples will be collected and embedded in RNA stabilization reagent for quantification of mRNA expression. However, the collection of paraffin embedded samples alone is sufficient to participate in the study. Tissue samples are collected strictly after TNM staging and patients with no leftover tumor tissue thereafter have to be excluded from the study. Likewise, patients with other final histological findings than PDAC cannot proceed within the study protocol. If the margins of the resected specimen show tumor cells when analyzed under the microscope (R1 resection), the patient can stay in the study as this population might still benefit from surgery and additional biomarker driven therapy in the future.

**Fig. 1:**
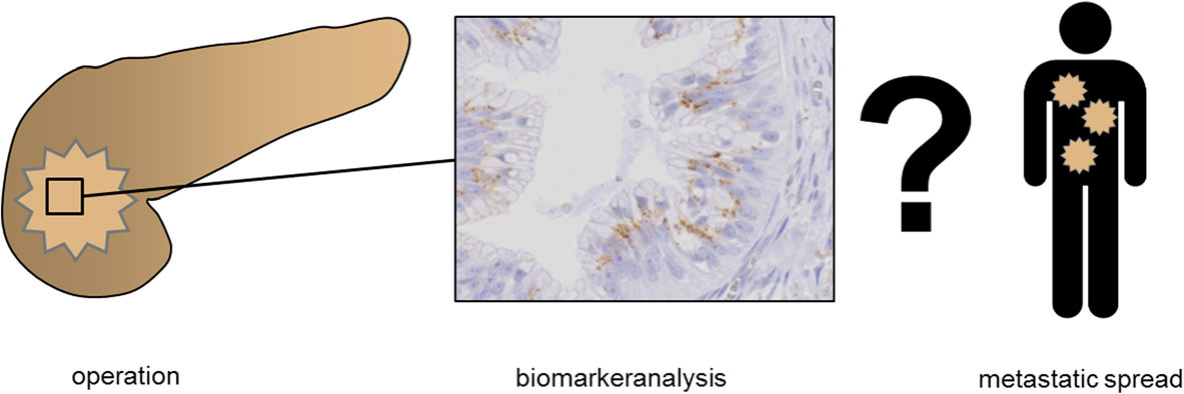
Design of the PANCALYZE trial: Tumor tissue samples are collected during surgery. Biomarker expression of CXCR4, SMAD4, SOX9 and IFIT3 will be assessed using immunohistochemistry. The patients are followed up to analyze if CXCR4^+^SMAD4^−^SOX9^+^IFIT3^+^ tumors will develop metastatic spread

Immunohistochemistry will be prospectively performed to assess biomarker expression of the tumors. This biomarker analysis will be correlated with the pattern of recurrence of the corresponding patients. The requisite clinical data will be collected over a 2-year follow-up observation period every 6 month by the coordinating center (see Table 2). Thus, it is possible to follow the individual patient’s clinical course and test if patients with CXCR4^+^SMAD4^−^SOX9^+^IFIT3^+^ tumors will develop metastatic spread (see Fig. 1).

**Table 2:** Schedule of enrolment, interventions and assessments (the responsible person is stated in brackets). As resectability of the tumor is a prerequisite to obtain tissue samples, the day of surgery (day 0) is the time point of allocation. Day of discharge (dis). Month (m)

	STUDY PERIOD							
	Enrolment	Allocation	Post-allocation					Close-out
TIMEPOINT	*until − 1*	0	*0*	*dis*	*6 m*	*12 m*	*18 m*	*24 m*
ENROLMENT:								
Eligibility screen *(surgeon)*	X							
Informed consent *(surgeon)*	X							
Allocation *(surgeon)*		X						
INTERVENTIONS:								
*Take 15 ml blood sample* *(surgeon)*			X					
*Prepare tumor tissue samples (pathologist)*			X					
ASSESSMENTS:								
*Diagnosis of PDAC* *(surgeon)*	X							
*Demographic data, operation procedure, complications*				X				
*Recurrence pattern, survival time, adjuvant tumor therapy and hospital admissions*					X	X	X	X

Ethical approval was obtained from the ethics committee of the University Hospital Cologne. Ethical approval was also obtained from the regional medical board’s ethics committees of the following German federal states: Sachsen-Anhalt, Sachsen, Brandenburg, Baden-Württemberg, Niedersachsen, Mecklenburg-Vorpommern. The study is registered at the German Clinical Trials Register (DRKS00006179, see Table 1).

## Biomarkers of the PANCALYZE study

### CXCR4

C-X-C chemokine receptor type 4 (CXCR4) is a transmembrane G-protein-coupled receptor which is expressed on cells of the immune system and on cancer cells. In contrast to other members of its family CXCR4 is activated by a single known ligand: CXCL12 which is also known as stromal-derived factor-1 (SDF-1). CXCL12 is highly expressed in lymph nodes, lung, liver and bone marrow and the CXCR4-CXCL12 axis plays an important role for stem cell homing to the bone marrow and the migration of leukocytes via chemotaxis - the directional migration of cells toward the higher concentration of the chemokine. Anti-CXCR4 blocking antibodies are explored to mobilize hematopoietic stem cells for bone marrow transplantation [8]. Acquirement of migratory functionality might play an important role in the development of metastasis: high expression of CXCR4 on cancer cells might induce their migration to tissue with high levels of CXCL12 [9]. CXCR4 expression has also been linked to over 20 different types of cancer like breast cancer [10], colon cancer [11], prostate cancer [12] and melanoma [13] demonstrating that the CXCR4/CXCL12-axis is involved in tumor progression, angiogenesis, metastasis and survival. In vitro and in vivo studies also have shown a correlation between tumor metastasis and alterations in CXCR4/CXCL12 signaling in PDAC [7, 12–16]. In a monocentric study high CXCR4 expression has found to be an independent prognostic biomarker in PDAC [17]. In the largest retrospective clinical study investigating CXCR4 including a total of 471 patients Bachet et al. described high CXCR4 expression to be a strong negative prognostic biomarker and predictor for the development of metastatic spread [6].

### SMAD4

The SMAD4 protein is a central mediator of the transforming-growth-factor β (TGF-β) signaling pathway. The TGF-β-family members regulate cell proliferation, differentiation and migration during embryonic development and tissue homeostasis in the adult. Aberrations in the signaling pathways have been linked to various diseases, such as autoimmune diseases, cardiovascular diseases and cancer. At autopsy Iacobuzio-Donahue et al. found loss of SMAD4 expression in only 22% of locally advanced carcinomas (*n* = 9) in contrast to 78% of patients with widespread metastatic disease (*n* = 22) [3]. Crane et al. showed in a phase II trial for locally advanced (T4) PDAC that 11 of 15 patients (73,3%) with intact SMAD4 expression suffered from local recurrence, and 10 of 14 Patients (71,4%) with loss of SMAD4 expression developed distant relapse [18]. However, there is diverging data concerning loss of SMAD4 expression as a predictor of survival and as a biomarker to predict the pattern of recurrence [3, 18]. In a multicenter study with 471 patients who underwent surgery for PDAC, SMAD4 evaluation did not correlate with recurrence pattern but hints on a survival benefit for adjuvant CTx [6]. Still, the huge amount of available data prompted us to include SMAD4 in the PANCALYZE trial, not least to resolve existing discrepancies.

### SOX9

SRY (sex determining region Y)-box 9 (SOX9) is a member of a high conserved family of transcription factors defined by their similarity to the “high mobility group” structure of the DNA binding domain. SOX9 plays an important role in sex determination, in chondrogenesis and in differentiation of multiple organs including normal pancreatic tissue [19]. Several studies have shown that SOX9 is differently expressed in many types of cancer, such as urothelial cancer [20], prostate cancer [21] and lung cancer [22] and is associated with tumor initiation and progression. Recently, Tanaka et al. investigated SOX9 expression in PDACs and in intraductal papillary mucinous neoplasms (IPMN). They found the rate of SOX9 positive cells was 82,7% in normal pancreatic tissue and 0,8% in PDAC. In IPMNs the rate of SOX9 positive cells decreased continuously with tumor progression [23]. In contrast to these findings recent studies demonstrated SOX9 overexpression in PDAC [24–27]. Kopp et al. identified SOX9 to be a critical mediator of acinar-to-ductal cell transformation which in turn is a principal mechanism of PDAC initiation [24]. SOX9 expression is associated with an unfavorable prognosis [28]. We have shown that inhibition of SOX9 expression in an orthotropic mouse PDAC tumor model leads to significant tumor size reduction, less angiogenesis, and fewer metastases [27]. Taken together SOX9 is a promising novel marker to be included into the PANCALYZE trial.

### IFIT3

Interferon-induced protein with tetratricopeptide repeats 3 (IFIT3) is one of numerous Interferon (IFN) stimulated genes (ISGs). Four canonical IFIT family members have been identified in humans- IFIT1, IFIT2, IFIT3 and IFIT5- that play important roles as effector molecules of the innate immune system. IFITs reside in the cytoplasm of host cells and can directly recognize viral specific RNA signature molecules [29]. Thereby they can inhibit distinct steps in the translation of viral RNA [30]. The role of IFIT3 in cancer is not well understood but inflammation is linked to pancreatic cancer progression and treatment-resistant metastasis. We have found that overexpression of IFIT3 enhances tumor growth, angiogenesis, metastasis and chemoresistance in vitro and therefore IFIT3 seems to be a tumor promoting protein. Moreover, IFIT3 over-expression was associated with poor prognosis in an explorative tissue microarray analysis of 250 human PDAC samples (unpublished observation). To complement these interesting data we included IFIT3 into our study analysis.

### Tissue sample analysis

Biomarker expression will be evaluated semi-quantitatively using standard immunochemistry. CXCR4 expression will be classified on a scale from 0 to 3, SMAD4 from 0 to 1, SOX9 from 0 to 2 and IFIT3 from 0 to 1. Samples will be examined by observers that are blinded to both clinicopathological data and the clinical course of the patients. Intra-observer and inter-observer variability will be controlled by multiple evaluations of the same sample by a single observer and by two independent trained observers evaluating the same sample, respectively. The grading of all four biomarkers will be combined into a single score (the PANCALYZE-score) as follows:

PANCALYZE-score = CXCR4 + 3*(1-SMAD4) + 3/2* SOX9 + 3*IFIT3.

Thus the PANCALYZE-score can be a number between 0 and 12 and represents the combined biomarker expression analysis. The PANCALYZE-score is modelled to normalize the semi-quantitative biomarker analysis. The weight factors ensure that all 4 biomarkers contribute to the PANCALYZE-score equally. The factors can be adjusted if one biomarker turns out to be significantly stronger compared to the others.

We expect to collect fewer samples for quantification of RNA expression fixed in RNAlater® because of the higher technical complexity to freeze and store these samples at the participating centers. These samples are used to confirm immunohistochemistry analysis using reverse transcription quantitative polymerase chain reaction (RT-qPCR) as a second method to analyze CXCR4, SMAD4, SOX9 and IFIT3 mRNA expression of the tumor.

### Data management

Documentation of demographic data, the operation procedure and complications during the hospital stay is accomplished by the surgeon of the participating center until the day of discharge. Follow-up assessing recurrence pattern, survival time, adjuvant tumor therapy and hospital admissions is performed by telephone interview with the attending oncologist by the coordinating center (see Table 2). Patients have to give informed consent as personal patient data must be stored non-encrypted to conduct follow-up. For analysis personal data will be pseudonymized. To collect data with minimal training of participating study centers we developed electronic forms for hospital data assessment as well as follow-up data assessment in portable document format (PDF, forms in German can be requested from the contact for scientific queries). PDF forms are user-friendly to handle and require no additional IT-infrastructure to be implemented in participating centers. They can be distributed conveniently to participating hospitals and sent back securely to the coordinating center (University Hospital Cologne). Data will be extracted from PDF files and stored in a central data base (MySQL, Oracle, Redwood City) in Cologne. This data base is managed by the institute for Quality Control in Operative Medicine which also monitors data quality independently from the sponsor. The final dataset can be accessed by the authors. Other researchers can apply for tissue samples and the dataset. The steering committee decides on a case by case basis if this scientific request is accepted.

### Statistical analysis and sample size calculation

The following variables will be collected for each patient: patient demographics, CA19-9, CEA, tumor characteristics (size, stage, grade, pathologic margin status, lymph node metastasis, lymphovascular invasion and perineural invasion). One-way ANOVA analysis will be used to identify new and published risk factors for metastatic spread and local recurrence [31]. To address the influence of adjuvant chemotherapy several subgroups will be defined: gemcitabine alone, gemcitabine and nab-paclitaxel, 5-fluorouracil based and others. As chemo- and radiotherapy might influence biomarker expression, we will analyze patients with locally advanced tumors receiving neoadjuvant therapy separately.

The PANCALYZE trail aims to develop a test to identify patients that develop metastatic spread and discriminate them from the other patients. Thus the PANCALYZE-score is a binary classifier. As no other test exists today we will conduct a phase I study. The receiver operating characteristic (ROC curve) is a well-established method to describe the performance of such a test. As the PANCALYZE-score is ordinal, ROC curves can be applied. To that end the PANCALYZE-score must be compared with the gold standard, the best possible method to identify patients with metastatic spread. Because the median survival of all patients is 21 months and we will follow the clinical course for at least 2 years it will be possible to exactly determine the pattern of recurrence of almost all patients. Thus ROC curve analysis will allow elucidating the performance of the PANCALYZE-score. Statistical analysis will be performed using the R environment for statistical computing (http://www.r-project.org/) and the package pROC [32].

Because this study is a phase I study there is no other test to compare the PANCALYZE-score to. Therefore the area under the ROC curve (AUC) serves as a parameter to qualify the test. To determine if the PANCALYZE-score can successfully discriminate patients with metastatic spread from the remaining patients the AUC must exceed 0.5. For the patient a far-reaching decision to conduct a non-standard therapy might rely on the quality of the test in the future. Therefore one would expect a better accuracy of the test demanding that the AUC exceeds 0.6. Assuming a significance level of 0.05 and a power of 0.95 a total of 410 patients have to be included into the study [33]. Secondary objectives are overall and disease free survival time which is measured from the date of surgery to the date of death.

## Discussion

The PANCALYZE trail aims to predict the clinical course of pancreatic cancer on the basis of CXCR4, SMAD4, SOX9 and IFIT3 expression. The biomarker analysis will be performed immediately after surgery to prospectively verify the hypothesis that patients with CXCR4^+^SMAD4^−^SOX9^+^IFIT3^+^ tumors most likely will develop metastatic spread.

The idea behind the PANCALYZE trail is that the pattern of propagation of pancreatic cancer is predefined by the genetic signature of the tumor. This hypothesis is strengthened by data from Iacobuzio-Donahue et al. [3] who observed 30% of patients with localized tumors versus 70% with metastatic disease at autopsy independent of the clinical stage at initial presentation. Today sophisticated tools like gene sequencing and DNA microarrays exist to analyze genetic signatures of tumors. However, these techniques require immense technical and financial efforts, which cannot be met in clinical daily routine and are limited to clinical trials today. With the help of cleverly selected biomarkers analyzed by immunohistochemistry the essential genetic signature defining tumor propagation might still be assessable. Immunohistochemistry can be performed in any hospital and might therefore be a valuable tool to predict the pattern of progression. However, immunochemistry is subject to the interpretation of the observer and interobserver error must be addressed in future studies.

We believe that one biomarker is not sufficient to encompass the complex mechanisms controlling tumor spread. Encouraging data from Bachet et al. [6] undermines the importance of CXCR4 expression for predicting disease prognosis and pattern of recurrence. However, CXCR4 as a single marker predicts the occurrence of metastatic spread with a sensitivity of 55% and a specificity of 63%. To deviate from current guidelines and give a patient a personalized different treatment according to biomarker analysis a more precise test is required. The combination of the 4 biomarkers is more robust and might be able to reliably predict the pattern of propagation.

The PANCALYZE study is a platform to test novel biomarkers. Additional biomarkers are evaluated constantly. If they have the potential to predict the pattern of recurrence they might be incorporated into the PANCALYZE-score. Promising biomarkers are best selected using genome wide transcriptome studies. Statistical analysis can reveal a few signature biomarkers describing the complex tumor biology. These are analyzed in the PANCALYZE-study using immunohistochemistry.

**Fig. 2:**
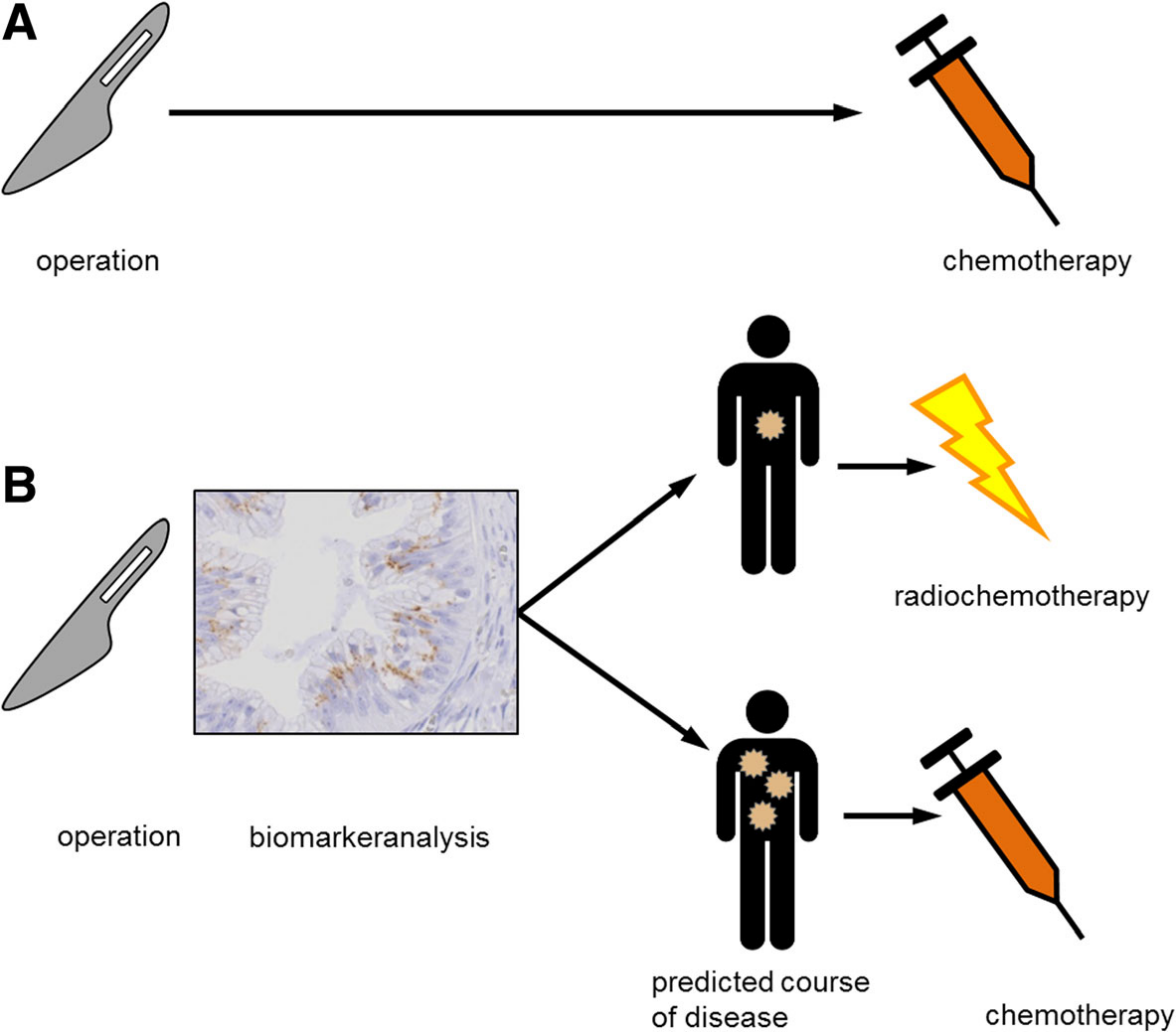
Future perspective: Today every patient receives adjuvant chemotherapy after surgical resection, regardless of individual tumor characteristics (**a**). If it is possible to predict the pattern of failure reliably, tumor treatment can be individualized and linked to biomarker expression patterns. Patients most likely developing metastatic spread might need intensified chemotherapy. Patients developing local recurrence might benefit from localized therapies like radiochemotherapy (**b**)

If our hypothesis is confirmed, future tumor treatment can be linked to biomarker expression patterns. Patients that most likely will experience metastatic spread after surgery might require intensified adjuvant CTx. Patients with localized disease that stays localized might benefit from additional local adjuvant treatment regimens like radiochemotherapy (see Fig. 2). If the basic pattern of propagation is predetermined by the biology of the tumor, surgery, CTx and radiochemotherapy most likely won’t change the pattern of failure fundamentally. In the future biomarker analysis can be extended and performed on tumor biopsies. Patients with a biomarker expression pattern indicating a high risk of developing metastases might not benefit from surgery at all. Biomarker analysis on biopsies may be used to identify personalized neoadjuvant therapies. Patients with a biomarker expression pattern associated with tumors that stay localized might benefit from neodjuvant radiochemotherapy even if the primary tumor is resectable.

In conclusion, biomarker analysis of a large quantity of pancreatic cancers as envisioned in the PANCALYZE trail will contribute to develop individualized treatment options in the future. Predicting the pattern of recurrence with the help of biomarkers is the first step toward personalized medicine for pancreatic cancer patients.

### Trail status

To recruit the 450 patients in short time, 14 hospitals from 6 different German states agreed to join the PANCALYZE trail. The study protocol was approved by the ethic committees of the University Hospital Cologne and of medical board’s ethics committees of 6 German federal states. Today, 14 hospitals already started to recruit patients. The current list of participants can be obtained by the primary sponsor (use contact for scientific queries). By the time of submission 39 patients were included into the study. The recruitment is planned to be completed until 2019. The follow-up will be performed until 2021.

## Abbreviations

AUC: area under the ROC curve
CTx: chemotherapy
C-X-C: chemokine receptor type 4 (CXCR4)
IFIT3: Interferon-induced protein with tetratricopeptide repeats 3
LAPC: locally advanced, unresectable non-metastatic pancreatic cancer
PDAC: Pancreatic ductal adenocarcinoma
PDF: portable document format
ROC curve: receiver operating characteristic
RT-qPCR: quantitative polymerase chain reaction
SOX9: sex determining region Y (SRY)-box 9
TGF-β: transforming-growth-factor β
